# Osteogenic commitment of Wharton’s jelly mesenchymal stromal cells: mechanisms and implications for bioprocess development and clinical application

**DOI:** 10.1186/s13287-019-1450-3

**Published:** 2019-11-28

**Authors:** Raquel Cabrera-Pérez, Marta Monguió-Tortajada, Ana Gámez-Valero, Raquel Rojas-Márquez, Francesc Enric Borràs, Santiago Roura, Joaquim Vives

**Affiliations:** 1 Cell Therapy Service, Blood and Tissue Bank (BST), Barcelona, Catalonia Spain; 2 Musculoskeletal Tissue Engineering Group, Vall d’Hebron Research Institute (VHIR) and Universitat Autònoma de Barcelona (UAB), Barcelona, Catalonia Spain; 3 REMAR-IVECAT Group, Health Science Research Institute Germans Trias i Pujol (IGTP), Badalona, Catalonia Spain; 4 Gene Regulation, Stem Cells and Cancer Program, Centre for Genomic Regulation (CRG), Barcelona, Catalonia Spain; 5 Nephrology Service, Germans Trias i Pujol University Hospital, Badalona, Catalonia Spain; 6 ICREC Research Program, Health Science Research Institute Germans Trias i Pujol (IGTP), Badalona, Catalonia Spain; 7 Medicine Department, Universitat Autònoma de Barcelona (UAB), Badalona, Catalonia Spain

**Keywords:** Mesenchymal stromal cells, Bone marrow, Wharton’s jelly, Osteogenic differentiation, Bone regeneration

## Abstract

**Background:**

Orthopaedic diseases are one of the major targets for regenerative medicine. In this context, Wharton’s jelly (WJ) is an alternative source to bone marrow (BM) for allogeneic transplantation since its isolation does not require an invasive procedure for cell collection and does not raise major ethical concerns. However, the osteogenic capacity of human WJ-derived multipotent mesenchymal stromal cells (MSC) remains unclear.

**Methods:**

Here, we compared the baseline osteogenic potential of MSC from WJ and BM cell sources by cytological staining, quantitative real-time PCR and proteomic analysis, and assessed chemical and biological strategies for priming undifferentiated WJ-MSC. Concretely, different inhibitors/activators of the TGFβ1-BMP2 signalling pathway as well as the secretome of differentiating BM-MSC were tested.

**Results:**

Cytochemical staining as well as gene expression and proteomic analysis revealed that osteogenic commitment was poor in WJ-MSC. However, stimulation of the BMP2 pathway with BMP2 plus tanshinone IIA and the addition of extracellular vesicles or protein-enriched preparations from differentiating BM-MSC enhanced WJ-MSC osteogenesis. Furthermore, greater outcome was obtained with the use of conditioned media from differentiating BM-MSC.

**Conclusions:**

Altogether, our results point to the use of master banks of WJ-MSC as a valuable alternative to BM-MSC for orthopaedic conditions.

## Background

The development of new treatments for bone-related diseases resulting from trauma or pathophysiological age-, sex- or infection-associated bone resorption has become a priority in the field of regenerative medicine [1–4]. In this context, autologous cell-based therapy has been presented as a promising approach to promote bone regeneration in both pre-clinical and clinical settings [5–8]. However, their clinical translation needs the delivery of safe and efficacious products, which can be largely hampered by age and co-morbidities of the cell donor [9–13]. In contrast, allogeneic off-the-shelf cell products derived from healthy and immune-compatible donors are very attractive since they are immediately available and provide a high number of cells [14, 15].

Multipotent mesenchymal stromal cells (MSC) constitute a heterogeneous population of non-haematopoietic multipotent cells which can be isolated from a variety of human body sources [3, 16, 17]. In particular, MSC have a fibroblast-like appearance, plastic adherence, the ability to differentiate into tissues of mesodermal lineages (adipocytes, chondrocytes and osteocytes) and a specific cell surface expression pattern, according to the minimal criteria established by the International Society for Cell and Gene Therapy (ISCT) [18]. Of note, a couple of MSC-based products have already received marketing approval [19], whereas most developments are still in clinical evaluation, including MSC-based tissue engineering products (EudraCT Nos. 2010-024041-78, 2010-023998-18, 2010-023999-12 and 2013-005025-23) under development in our laboratory [20].

Bone marrow (BM) has become the most used source of MSC in the orthopaedic field because of its intrinsic osteogenic differentiation potential, but alternative sources are garnering attraction. These include Wharton’s jelly (WJ), which is the connective tissue surrounding the human umbilical cord and is advantageous for cell collection since its isolation is not painful, does not require invasive procedures and does not raise major ethical concerns [21]. Accordingly, we reported the feasibility of expanding clinical-grade WJ-MSC from samples typically discarded from public cord blood banking programmes [22]. Moreover, in contrast to adult BM-MSC, WJ-MSC are expected to be more primitive, proliferative and immunosuppressive cells, particularly for the lack of HLA-DR antigens [23–26]. Nevertheless, the osteogenic capacity of WJ-MSC remains under scrutiny.

Osteogenic differentiation of mesenchymal precursors and bone regeneration are extremely complex processes regulated by the interaction of different signalling pathways including TGFβ/BMP, MAPK, Wnt, Hedgehog, Notch and AKT/mTOR [27]. Among them, the TGFβ/BMP pathway plays the major role in the regulation of osteoblast lineage-specific differentiation, bone induction, maintenance and repair and constitutes a promising target for the treatment of bone diseases [28–30]. Up to date, about 60 TGFβ family proteins have been identified so far, being TGFβ1 and BMP2 ligands the most widely investigated due to their positive role in bone formation in vivo [31, 32]. However, while BMP proteins have been demonstrated to induce the expression of MSC differentiation factors (such as *DLX5* and *RUNX2*) [33, 34], several in vitro studies have described a negative impact of TGFβ1 on the terminal differentiation of osteoblast precursors [34–36].

Despite several reports investigating the osteogenic capabilities of BM- and WJ-MSC have been published to date, it is still difficult to integrate existing data due to heterogeneity in MSC isolation and culture procedures. In the present study, we aimed to provide homogeneous and comparative data regarding the ability of BM- and WJ-MSC to differentiate towards the osteogenic lineage by means of cytological staining and molecular and proteomic analysis. Moreover, we evaluated a variety of strategies based on the modulation of the TGFβ/BMP pathway and the use of the BM-MSC secretome to enhance osteogenesis in WJ-MSC and emulate BM-MSC osteogenic commitment.

## Methods

### Cell culture

BM-MSC (*n* = 3) and WJ-MSC (*n* = 3) (passage 3–5) were isolated according to ‘Good Manufacturing Practice for Advanced Therapy Medicinal Products’ (GMP for ATMPs, European Commission Guidelines of 2017.11.22) and further expanded in Dulbecco’s modified Eagle’s medium (DMEM) (31885-023; Gibco) containing 2 mM glutamine and supplemented with 10% human serum B (hSerB)—‘expansion medium’ [37, 38]. All cell cultures were maintained at 37 °C and 5% CO_2_ in humidified incubators, and media were changed every 3–4 days. Cell number and viability were determined by the haemocytometer-based trypan blue dye exclusion assay.

### Phenotype assessment

Immunophenotypic characterisation of MSC was performed using the following antibodies: mouse anti-human CD45-fluorescein isothiocyanate (CD45-FITC) (Clone HI30; 555482; BD Pharmingen), anti-human CD105-phycoerythrin (CD105-PE) (Clone 43A4E1; 130-117-696; Miltenyi Biotec), anti-human HLA-DR-FITC (Clone L243; 347363; BD Biosciences), anti-human CD90-PE (Clone F15-42-1-5; IM1840U; Beckman Coulter), anti-human CD31-FITC (Clone WM59; 555445; BD Pharmingen) and anti-human CD73-PE (Clone AD2; 550257; BD Pharmingen). Cells were stained for 15 min at room temperature (RT), washed and re-suspended in phosphate-buffered saline (PBS) (14190-094; Gibco) as described elsewhere [38]. Acquisition and data analysis were performed using a FACSCalibur cytometer and the CellQuest Pro software (Becton Dickinson), respectively.

### Osteogenic differentiation assays

BM-MSC and WJ-MSC (passage 3–5) were seeded until 70–80% confluence (10^4^ and 2 × 10^4^ cells/cm^2^, respectively). ‘Differentiation media’ composed of the StemPro osteogenesis differentiation kit (A1007201; Gibco) supplemented with 100 units/mL of penicillin and 100 μg/mL streptomycin (Penicillin-Streptomycin; P4458; Sigma-Aldrich) was used for the osteogenic induction in vitro. Alkaline phosphatase (ALP) (B5655; Sigma-Aldrich) and alizarin red (AR) (2003999; Merck Millipore) staining were finally carried out to assess cell differentiation.

### Gene expression assays

Total RNA was purified from cell cultures using the RNeasy Plus Mini Kit (74134; Qiagen) according to the manufacturer’s instructions, quantified using NanoDrop Lite (Thermo Scientific), and electrophoresed in 1% agarose gels to confirm integrity and purity. cDNA synthesis was then performed by reverse-transcription PCR (RT-PCR) using the High-Capacity cDNA Reverse Transcription Kit (4368814; Thermo Fisher Scientific) according to the manufacturer’s instructions. cDNA was finally amplified by quantitative real-time PCR (qRT-PCR) using the TaqMan gene expression assays listed in Table 1. In all cases, target gene expression was referred to *GAPDH* expression by using the 2^−ΔCt^ method.

**Table 1:** TaqMan gene expression assay ID

Gene	Assay ID
*MSX2*	Hs00741177_m1
*DLX5*	Hs01573641_mH
*RUNX2*	Hs01047973_m1
*SP7*	Hs05049492_s1
*ALPL*	Hs01029144_m1
*TFGβ1*	Hs00998133_m1
*BGLAP*	Hs01587814_g1
*COL1A2*	Hs01028970_m1
*GAPDH*	Hs02786624_g1

### Inhibition/activation of TGFβ/BMP2 signalling pathways

WJ-MSC (passage 4) were seeded at a cell density of 2 × 10^4^ cells/cm^2^. Inhibition of the TGFβ signalling pathway was carried out by addition of galunisertib (LY2157299) (sc-391123; Santa Cruz Biotechnology) to the osteogenic differentiation media at a final concentration of 10 μM. For BMP2 pathway stimulation, human recombinant BMP2 (SRP6155; Sigma-Aldrich) and/or tanshinone IIA (sc-200932; Santa Cruz Biotechnology) were added to a final concentration of 100 ng/mL and 5 μM, respectively. All compounds were added immediately after every media change after 1 week of in vitro osteogenic induction with differentiation media.

### Extracellular vesicle isolation and characterisation

To avoid sample contamination with exogenous extracellular vesicles (EVs), cells were cultured in EV-depleted media. For EV depletion, 2× differentiation media was ultra-centrifuged at 100,000 × *g* for ≥16 h and diluted with StemPro Basal Media (A10069-01; Gibco) to 1× working concentration.

Supernatants derived from undifferentiated BM-MSC and WJ-MSC (passage 3–5) or from BM-MSC under osteogenic differentiation (passage 3–5) were collected at weeks 0, 1, 2 and 3, and were sequentially centrifuged at 400 × *g* for 5 min and at 2000 × *g* for 10 min to exclude cells and cell debris. Conditioned medium (CM) was then concentrated by 100-kDa ultrafiltration using Amicon Ultra (UFC910024; Millipore) at 2000 × *g* for 35 min, obtaining typically 250 μL of concentrated CM (CCM). EVs were isolated by size exclusion chromatography (SEC) as previously reported [39]. Protein elution was checked by reading absorbance at 280 nm using NanoDrop (Thermo Scientific).

The presence of EVs in the SEC fractions was determined according to the presence of tetraspanins by bead-based flow cytometry [39]. Briefly, EVs were coupled to 4-μm aldehyde/sulphate-latex microspheres (A37304; Invitrogen) for 15 min at RT and blocked in BCB buffer (PBS supplemented with 0.1% BSA (A4503) and 0.01% NaN_3_ (S8032); Sigma-Aldrich) on overnight rotation. EV-coated beads were spun down at 2000 × *g* for 10 min, washed with BCB buffer and re-suspended in PBS. EV-coated beads were labelled with the primary antibodies anti-CD9 (Clone VJ1/20) and anti-CD63 (Clone TEA3/18) (kindly provided by M. Yáñez-Mó (CBM-SO, IIS-IP, UAM, Madrid, Spain) and F. Sánchez-Madrid (Hospital Universitario de la Princesa, IIS-IP, UAM, CNIC, Madrid, Spain)) or the IgG isotype control (a637355; Abcam) and secondary antibody FITC-conjugated Goat F(ab′)2 Anti-Mouse IgG (1032-02; Bionova). EV-coupled beads were washed after each step with BCB buffer and centrifuged at 2000 × *g* for 10 min. Data was acquired in a FACSLyric flow cytometer (BD) and analysed by FlowJo v.X software (Tree Star).

SEC-EV-containing fractions were examined for EV size and morphology by cryo-electron microscopy (cryo-EM). Vitrified specimens were prepared by placing 3 μL of a sample on a Quantifoil® 1.2/1.3 TEM grid, blotted to a thin film and plunged into liquid ethane-N2_(l)_ in the Leica EM CPC cryoworkstation (Leica). The grids were transferred to a 626 Gatan cryoholder and maintained at −179 °C. Samples were analysed with a Jeol JEM-2011 transmission electron microscope (Jeol) operating at an accelerating voltage of 200 kV. Images were recorded on a Gatan UltraScan 2000 cooled charge-coupled device (CCD) camera with the DigitalMicrograph software package (Gatan).

### Proteomic analysis

The protein content of EV-enriched fractions was analysed by liquid chromatography followed by mass spectrometry (LC-MS/MS) on Orbitrap XL (Thermo Fisher) for three independent undifferentiated cultures for each MSC type. Data was searched against the Swiss-Prot human database (downloaded in August 2016), using the search algorithm Mascot v2.5.1. Only peptides showing a false discovery rate (FDR) lower than 5% were retained. Proteins identified with at least two unique peptides and found in all three samples were considered for further analysis.

The obtained proteomic profile for our samples was compared with previous studies compiled in EV-specific databases EVpedia [40], ExoCarta [41] and Vesiclepedia [42].

### Data analysis

Statistical analysis was performed with the GraphPad Prism 6 software (GraphPad Software, Inc.). Descriptive data were expressed as mean ± standard deviation (SD). Multiple *t* tests were used for investigating differences between BM- and WJ-MSC at different time points along the osteogenic differentiation. Statistical significance was set at *p < 0.05 and **p < 0.01.

## Results

### WJ-MSC exhibit delayed osteogenic induction compared with BM-MSC

BM-MSC and WJ-MSC were highly positive for CD73, CD90 and CD105 and negative for CD31, CD45 and HLA-DR expression according to the ISCT criteria (Additional file 1).

**Fig. 1:**
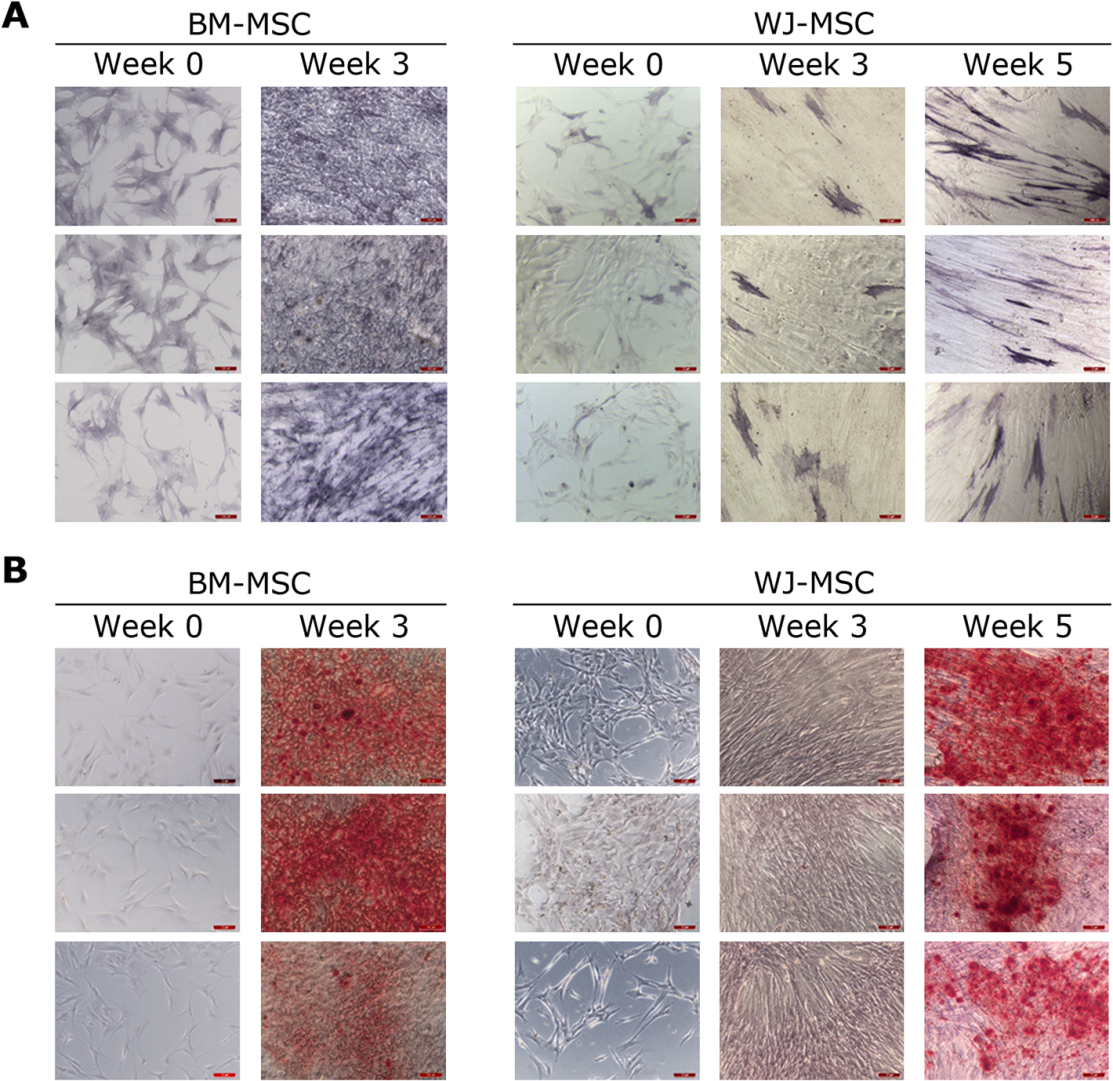
Differences in timing for osteogenic differentiation in BM-MSC and WJ-MSC cultures. Alkaline phosphatase (**a**) and alizarin red (**b**) staining at indicated times. *N* = 3 for both MSC types. Scale bars: 100 μm

The osteogenic differentiation of BM-MSC and WJ-MSC cultures were assessed by alkaline phosphatase (ALP) and alizarin red (AR) staining. All BM-MSC and WJ-MSC cell lines showed osteogenic potential in vitro after osteogenic induction with specific differentiation media. However, a delay in osteogenesis was observed in WJ-MSC compared to BM-MSC. In particular, as shown in Fig. 1a, most of the cells in BM-MSC cultures displayed a marked baseline activity of the osteogenic marker ALP. In contrast, in WJ-MSC cultures, only few cells exhibited ALP activity even at week 5. Regarding the results obtained for AR staining, calcium depositions were clearly visible in BM-MSC cultures at week 3. However, 5 weeks were required in order to obtain similar results in WJ-MSC (Fig. 1b).

### BM-MSC show increased expression of osteogenic markers compared to WJ-MSC

**Fig. 2:**
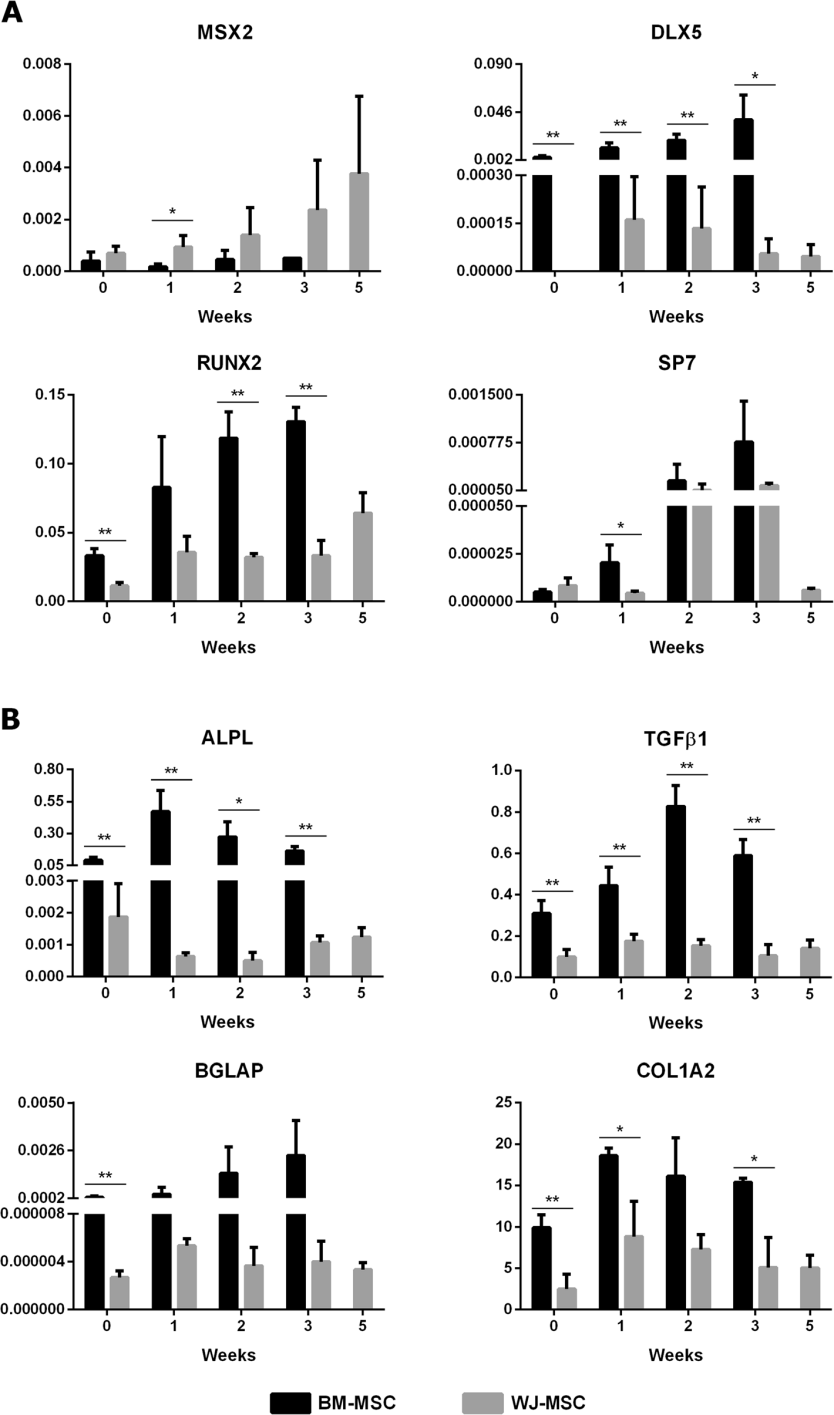
Gene expression profiles of the main markers involved in osteogenic differentiation. Expression levels of osteogenic transcription factors (**a**) and early/late osteogenic markers (**b**). Bars represent mean ± SD. *p < 0.05 and **p < 0.01 (multiple *t* tests). *N* = 3 for each MSC type

We further analysed the delayed osteogenesis in WJ-MSC cultures. For that purpose, we comparatively assessed the time-course expression of key osteogenic genes implicated in MSC differentiation. Interestingly, we found some significant differences along both BM- and WJ-MSC osteogenic differentiation. Regarding osteogenic transcription factors (Fig. 2a), BM-MSC showed a progressive increase in *DLX5*, *RUNX2* and *SP7* expression. In contrast, in WJ-MSC, *DLX5* expression was gradually decreased and *SP7* levels exhibited an increment from week 2 to 5*.* Additional differences were observed in *MSX2*. Remarkably, a steady increase of *MSX2* expression was observed up to week 5 in WJ-MSC, whereas no changes in *MSX2* expression were detected in BM-MSC.

Moreover, the expression patterns of main early and late osteogenic markers were comparatively assessed (Fig. 2b). In this sense, the early marker *ALPL* reached its maximum expression level during the first week in BM-MSC. However, in WJ-MSC, *ALPL* expression was reduced at this time compared to week 0 and started to increase again from the third week. The expression patterns of late osteogenic markers *TGFβ1* and *BGLAP* were also different. In BM-MSC, *TGFβ1* achieved the highest expression level at week 2 and *BGLAP* expression increased progressively. On the contrary, no changes were observed for these genes in WJ-MSC. In regard to *COL1A2*, similar expression patterns were obtained for both cell types, although expression in BM-MSC was twofold higher than that in WJ-MSC. Remarkably, the expression of *RUNX2*, *DLX5*, *ALPL*, *TGFβ1*, *BGLAP* and *COL1A2* was promoted in BM-MSC even when they were in an undifferentiated stage. Taken together, these findings indicate a higher osteogenic differentiation commitment in BM-MSC.

### Promotion of BMP2 signalling primes osteogenic differentiation of WJ-MSC

**Fig. 3:**
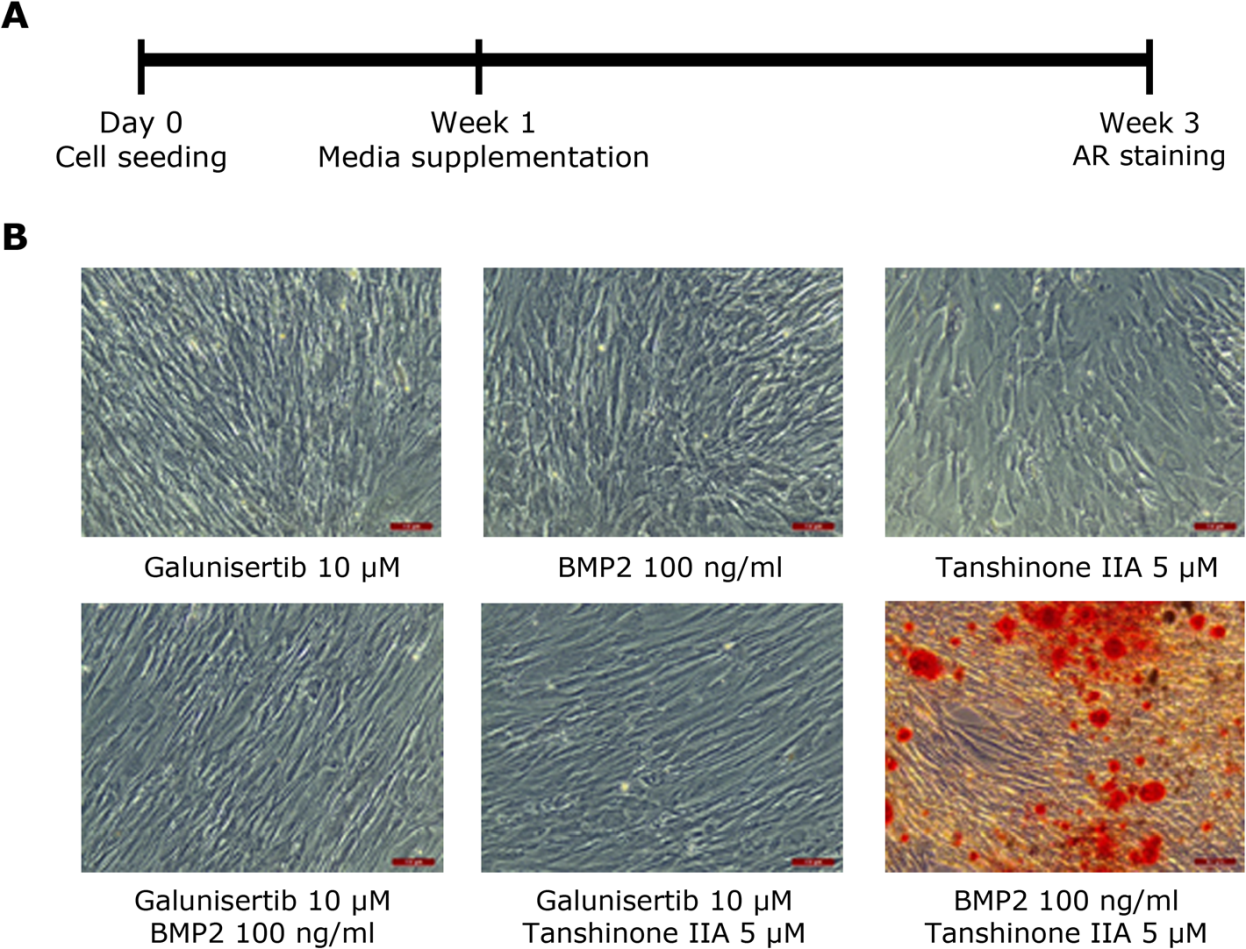
Modulation of the TGFβ/BMP signalling pathway to stimulate WJ-MSC osteogenic differentiation. **a** Scheme of the experimental design. From day 0 to week 1, cells were cultured in osteogenic differentiation media, which was supplemented from week 1 to 3 with galunisertib, BMP2 and/or tanshinone IIA. **b** Representative AR staining results obtained in passage 4 WJ-MSC after 2 weeks of culture in osteogenic media supplemented with galunisertib 10 μM, BMP2 100 ng/mL and tanshinone IIa 5 μM in different combinations. Scale bars: 100 μm

Subsequently, the role of TGFβ1 and BMP2 signalling pathways in the promotion of osteogenic differentiation of WJ-MSC was investigated. To prevent TGFβ activation, differentiation media was supplemented from week 1 to 3 with galunisertib. On the other hand, in order to stimulate osteogenic differentiation through the BMP2 signalling pathway, human recombinant BMP2 and/or the BMP activator tanshinone IIA were also added to the differentiation media (Fig. 3a).

As shown in Fig. 3b, the inhibition of TGFβ1 signalling had no effect on WJ-MSC osteogenesis. Similarly, negative results in AR staining experiments were seen when BMP2 and tanshinone IIA were present alone or in combination with galunisertib. In contrast, when BMP2 and tanshinone IIA were added together, calcium depositions were clearly visible in WJ-MSC cultures after 3-week induction, emulating the behaviour of BM-MSC under standard osteogenic inducing conditions. This suggests the promotion of WJ-MSC osteogenic differentiation following stimulation of BMP2 signalling.

### BM-MSC-conditioned media strongly enhances WJ-MSC osteogenic differentiation

**Fig. 4:**
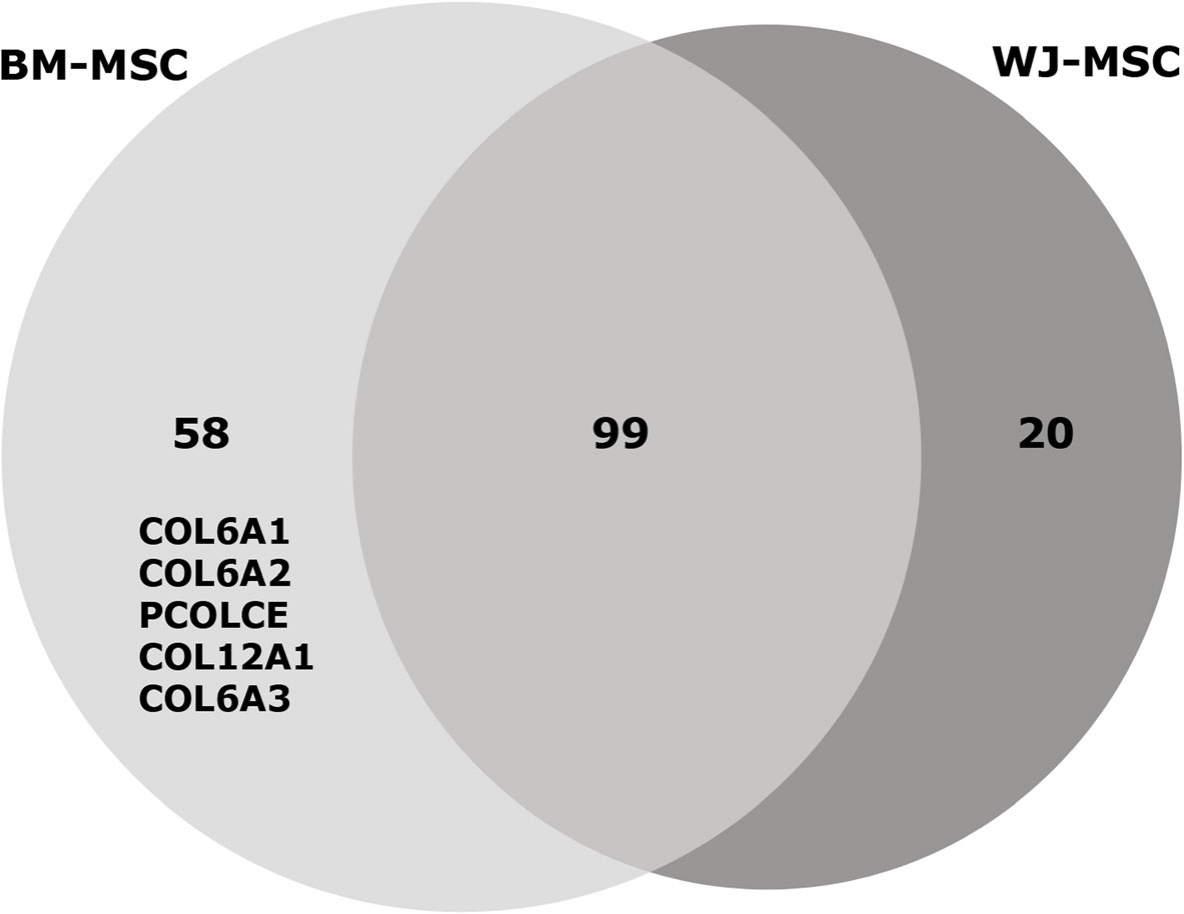
Osteogenic markers found in undifferentiated BM-MSC- and WJ-MSC-secreted EVs. Number of proteins found in extracellular vesicles (EVs) isolated by size exclusion chromatography (*n* = 3 different cell lines for BM-MSC and WJ-MSC). The osteogenic markers differentially overrepresented in BM-MSC EV samples are indicated. Results were obtained by LC-MS/MS, two peptides restricted, FDR<5%

The results obtained after the characterisation of the gene expression profiles of some of the main osteogenic markers explained, in part, the superior osteogenic potential presented by BM-MSC. However, with the aim of deepening in the elucidation of the increased osteogenic capacity shown by BM-MSC, the proteomic content associated with the isolated extracellular vesicles (EVs) from the supernatant of undifferentiated BM-MSC and WJ-MSC cultures was analysed. Proteomic analysis by LC-MS/MS allowed the identification of several EV markers, such as annexin A2, A5 and A6, glyceraldehyde-3-phosphate dehydrogenase and CD5L. As depicted in Fig. 4, 99 proteins were found in common between both types of cells. However, five osteogenic markers (namely COL6A1, COL6A2, PCOLCE, COL12A1 and COL6A3) were differentially overrepresented in BM-derived EVs compared to WJ-MSC EVs. This finding could contribute to the explanation of the higher osteogenic commitment observed in BM-MSC and suggested the possibility of using the BM-MSC secretome to prime osteogenesis in WJ-MSC.

To test the feasibility of this approach, conditioned media (CM) from BM-MSC cultures were obtained weekly up to week 3 of the osteogenic differentiation and processed by size exclusion chromatography to separate and purify fractions enriched in EVs or soluble proteins (Additional file 2). Once isolated, the EV or protein fractions were added to WJ-MSC differentiating cultures.

**Fig. 5:**
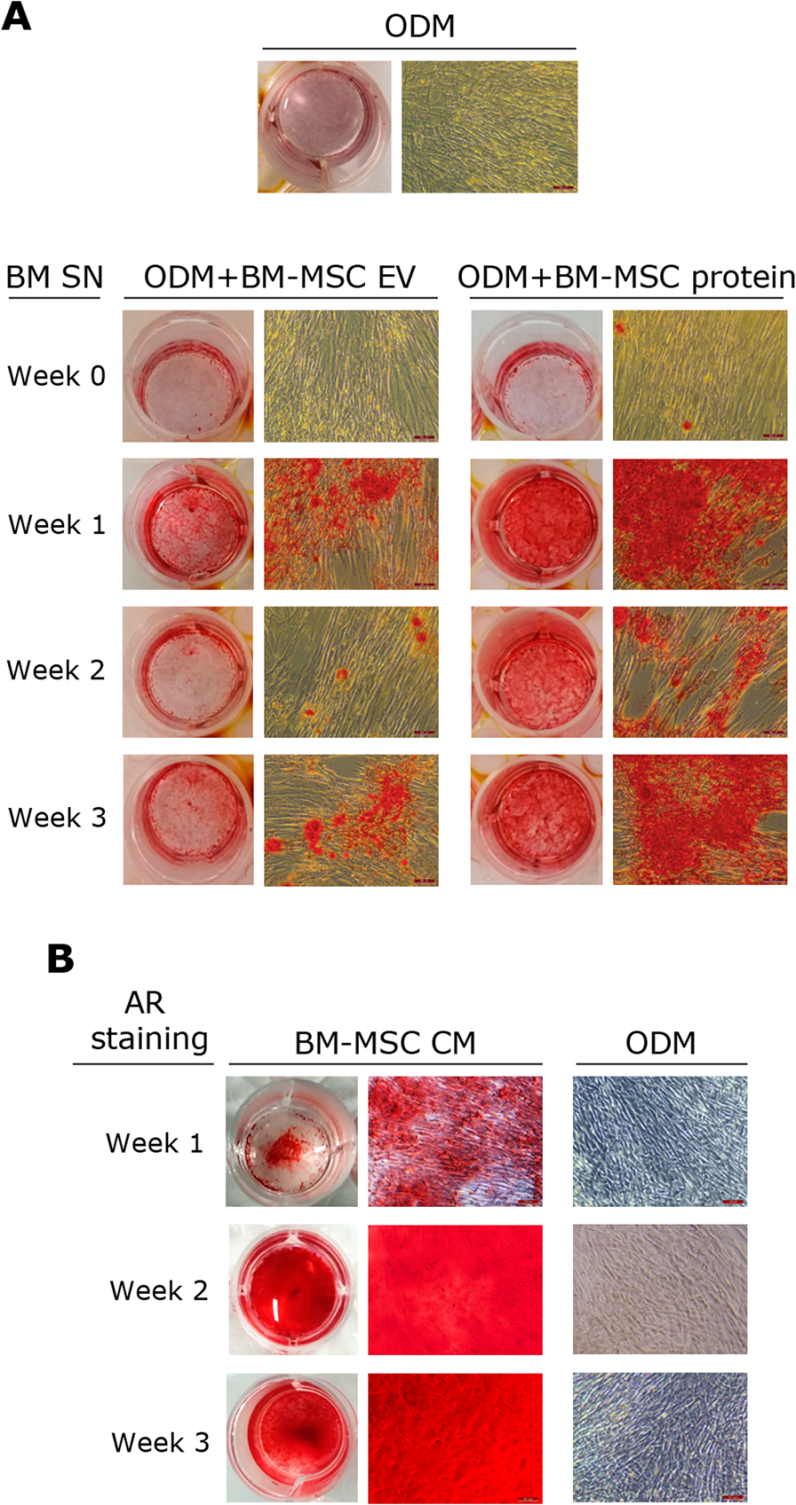
Effect of BM-MSC-derived products on osteogenic commitment of WJ-MSC. **a** Representative images of AR staining in passage 4 WJ-MSC after 3 weeks of culture with ODM alone (top) or supplemented with either EV or protein fractions collected from undifferentiated BM-MSC (week 0) or BM-MSC cultures at week 1, 2 or 3 of osteogenic differentiation (bottom). **b** Representative images of AR staining in passage 4 WJ-MSC after 1, 2 or 3 weeks of culture with CM obtained from differentiating BM-MSC cultures at week 1. CM conditioned media, EV extracellular vesicles, ODM osteogenic differentiation media, SN supernatant. In all cases, scale bars: 100 μm

Monitoring of the osteogenic differentiation was performed by AR staining. Negative results were obtained in all cases after 2 weeks of media supplementation with either EVs or protein fractions (data not shown). However, after 3 weeks, calcium depositions were observed in all the WJ-MSC cultures supplemented with BM-secreted EVs and protein fractions purified from BM-MSC differentiating cultures at weeks 1, 2 and 3, while the secretome from undifferentiated BM-MSC (week 0) did not (Fig. 5a). Although both EVs and protein fractions positively stimulate WJ-MSC osteogenesis, the effect produced by the soluble protein fraction resulted in a greater outcome than that produced by EVs collected at the same differentiation stage. Based on this observation, the full CM collected from BM-MSC cultures after 1 week of in vitro osteogenic induction was also tested (Fig. 5b). As shown in Fig. 5b, the addition of CM from differentiating BM-MSC to WJ-MSC cultures resulted in higher osteogenesis than the addition of purified EVs or protein fractions separately. Furthermore, the fact that calcium depositions were clearly identified from the very first week indicated a powerful synergistic positive effect between EV and proteins secreted by BM-MSC in the progression along the osteogenic linage differentiation.

## Discussion

The use of allogeneic MSC is promising for the treatment of bone-related conditions. In this context, some studies have suggested that master cell banks of WJ-MSC may offer advantages over the use of BM-MSC [20, 22]. However, to guarantee the success of WJ-MSC in situations where bone repair or bone regeneration is required, it is essential to demonstrate that osteogenic commitment is equally driven in both BM- and WJ-MSC.

In the present study, we have extensively characterised the osteogenic differentiation process of BM- and WJ-MSC in order to provide homogeneous data because, despite there are many reports in the field describing osteogenic properties of both cell lines, data sets are often incomplete and non-comparable due to heterogeneity in isolation and culture procedures.

In concordance with previous observations reported by other authors in the field [43, 44], our findings evidenced that WJ-MSC are less prone to differentiate into bone cells than BM-MSC. We thus aimed to comparatively analyse the molecular machinery associated with osteogenic differentiation in BM- and WJ-MSC since we found a marked delay in the osteogenic progression of WJ-MSC compared to BM-MSC. Results obtained by qRT-PCR confirmed that BM-MSC exhibit an osteogenic gene profile more similar to that of osteoblast and evidenced that MSX2, a TGFβ1-induced factor reported to promote cell proliferation and supress osteogenic differentiation by inhibiting DLX5-driven effects [36, 45–48], was overexpressed in WJ-MSC. This suggests that the balance between *MSX2* and *DLX5* expression could be critical in MSC, resulting in either a proliferating or differentiating outcome.

Trying to revert the scenario found in WJ-MSC, we then attempted to modulate TGFβ- and BMP-mediated signalling pathways by adding specific activators or inhibitors in order to repress *MSX2* and/or to stimulate *DLX5* expression. To this end, we used different combinations of chemical factors including (i) galunisertib, a receptor antagonist that specifically targets and binds TGFβRI [49]; (ii) BMP2, which has been reported to induce osteoblast differentiation by promoting *DLX5* expression [33, 50] and (iii) tanshinone IIA, a phytochemical compound reported to enhance BMP signalling stimulation [51]. Neither the use of each molecule separately nor the combination of a repressing and activating agent resulted in an osteogenic output in WJ-MSC. However, agreeing with the results published by Heo and collaborators in umbilical cord blood MSC [52], the addition of tanshinone IIA to BMP2-induced cultures significantly increased calcium depositions in WJ-MSC after 3 weeks, thus matching WJ-MSC osteogenic behaviour to that of their BM-MSC counterparts.

Although systemic infusion of MSC has been shown to increase bone growth and repair in clinical trials [53–55], administered cells engraft poorly. In this line, previous in vivo studies carried out by our group in which ovine *eGFP+* BM-MSC were infused in an ovine model of osteonecrosis of the femoral head demonstrated the presence of non-stained eGFP osteocytes in newly formed bone matrix, suggesting that contribution of MSC lies also in paracrine signalling that activate and recruit host osteoblasts to the bone repair areas [56]. Increasing evidence have shown that nanosized, membrane-encapsulated EVs are one of the most active MSCs’ secreted factors [25]. Indeed, EVs can serve as powerful tools for cell-free therapy due to precise multifunctional molecular cargoes [57, 58]. However, significant differences have been described in the content of EV purified from MSC cultures of different origins. In terms of miRNA profiles, BM-MSC-derived EVs have been described to present a miRNA cargo that is tightly related to MSC differentiation [59]. Furthermore, different profiles of miRNAs have also been reported depending on the differentiation stage of the secretory cell [60]. Here, we studied the differences in the protein content of EVs obtained from undifferentiated BM- and WJ-MSC. As a result, COL6A1, COL6A2, PCOLCE, COL12A1 and COL6A3 osteogenic markers were differentially overrepresented in BM-derived EVs compared to WJ-MSC EVs. Interestingly, COL6 and COL12 interactions have been reported to control and promote bone formation in early phases due to their role in the establishment of matrix bridges between adjacent cells when pre-osteoblasts establish cell-cell communication [61]. These observations prompted us to evaluate the effect of BM-MSC-derived EVs in the osteogenic differentiation of WJ-MSC. Additionally, the impact of the soluble protein fractions (eluted in the latter size exclusion chromatography (SEC) fractions) purified from BM-CM was also determined.

Both EV-rich and soluble protein fractions from differentiating BM-MSC cultures promote bone differentiation in WJ-MSC. On the contrary, the secretome of undifferentiated BM-MSC does not have an osteogenic effect. This fact matches the differences reported in the miRNA content of MSC-EVs depending on differentiation stages and explains, in part, the little differences found in the protein content of undifferentiated BM-MSC and WJ-MSC EVs. Remarkably, the use of unprocessed CM obtained from differentiating BM-MSC has a greater impact on osteogenic induction and produces not only an exacerbation on the mineralisation of the culture but also a shortening in the differentiation time. This suggests that EV- and protein-rich fractions from differentiating BM-MSC act by different and synergistic pathways, thus pointing out that BM-MSC and WJ-MSC therapeutic efficacy could be equivalent when administered within the bone microenvironment, where BM-MSC are present, and mitigating the need to overstimulate WJ-MSC osteogenesis ex vivo*.* This is particularly relevant due to the implications of WJ-MSC osteogenic priming in manufacturing development and clinical applications which include (i) prolonged cell cultures for ex vivo stimulation; (ii) difficulties of cell trypsinisation once the differentiation process is started; (iii) higher costs associated with the increment in time for cell culture and the use of additional GMP grade products; and (iv) possible clinical complications surrounding the use of priming compounds. Furthermore, the effect associated with the use of CM could be enhanced in vivo due to the immune response produced under pathophysiological conditions, which positively contributes to bone regeneration.

## Conclusions

Despite their multipotentiality, the intrinsic molecular signature of WJ-MSC described here highly counteracts their osteogenic differentiation and thus their future application in cell-based therapies against orthopaedic conditions. However, our findings demonstrate that secreted factors in the CM from differentiating BM-MSC cultures greatly enhance WJ-MSC osteogenesis and suggest that intra-bony environment could be enough to guarantee WJ-MSC-promoted bone regeneration. This fact avoids the need either to overstimulate WJ-MSC osteogenesis ex vivo or to use genetically modified WJ-MSC. Therefore, although further research is required in order to evaluate the therapeutic benefit of WJ-MSC in the context of orthopaedic diseases, the use of GMP-grade master cell banks of WJ-MSC may be a valuable alternative to those of BM-MSC.

## Supplementary information

**Additional file 1 **Immunophenotypic characterization of BM-MSC and WJ-MSC. Boxes represent median and 5-95 percentiles. *N* = 3 for each cell type.

**Additional file 2.** EV and protein fractions isolation and characterization. (A) Scheme of the methodological procedure followed for EV and protein isolation from BM-MSC and WJ-MSC conditioned media. CCM, concentrated conditioned media; CM, conditioned media; EV, extracellular vesicles; SEC, size exclusion chromatography; SN, cell culture supernatant. (B) Example of representative elution profile obtained for CD9 and CD63 EV markers quantification by bead-based flow cytometry (left axis) and for protein elution monitoring by absorption at 280 nm (right axis) in the different SEC fractions. MFI, mean fluorescence intensity. (C) Cryo-EM images confirming EVs presence in pooled EV fractions. Scale bar: 200 nm.

## Data Availability

The datasets used and/or analysed during the current study are available from the corresponding authors on request.

## Abbreviations

ALP: Alkaline phosphatase
AR: Alizarin red
BM: Bone marrow
CM: Conditioned media
EV: Extracellular vesicle
FDR: False discovery rate
GMP: Good manufacturing practice
ISCT: International Society for Cell and Gene Therapy
MSC: Mesenchymal stromal cell
ODM: Osteogenic differentiation media
qRT-PCR: Quantitative real-time PCR
RT: Room temperature
SEC: Size exclusion chromatography
WJ: Wharton’s jelly
