# Supplementary material for: Osteogenic commitment of Wharton’s jelly mesenchymal stromal cells: mechanisms and implications for bioprocess development and clinical application
Source: Stem Cell Res Ther. 2019 Nov 28;10:356. doi: 10.1186/s13287-019-1450-3 (PMC6883559; doi:10.1186/s13287-019-1450-3)
Supplement: Supplementary file 1 — Additional file 1 Immunophenotypic characterization of BM-MSC and WJ-MSC. Boxes represent median and 5-95 percentiles. N = 3 for each cell type. [file 13287_2019_1450_MOESM1_ESM.docx]

**ADDITIONAL INFORMATION**





**Additional file 1. Immunophenotypic characterization of BM-MSC and WJ-MSC.** Boxes represent median and 5-95 percentiles. N=3 for each cell type.
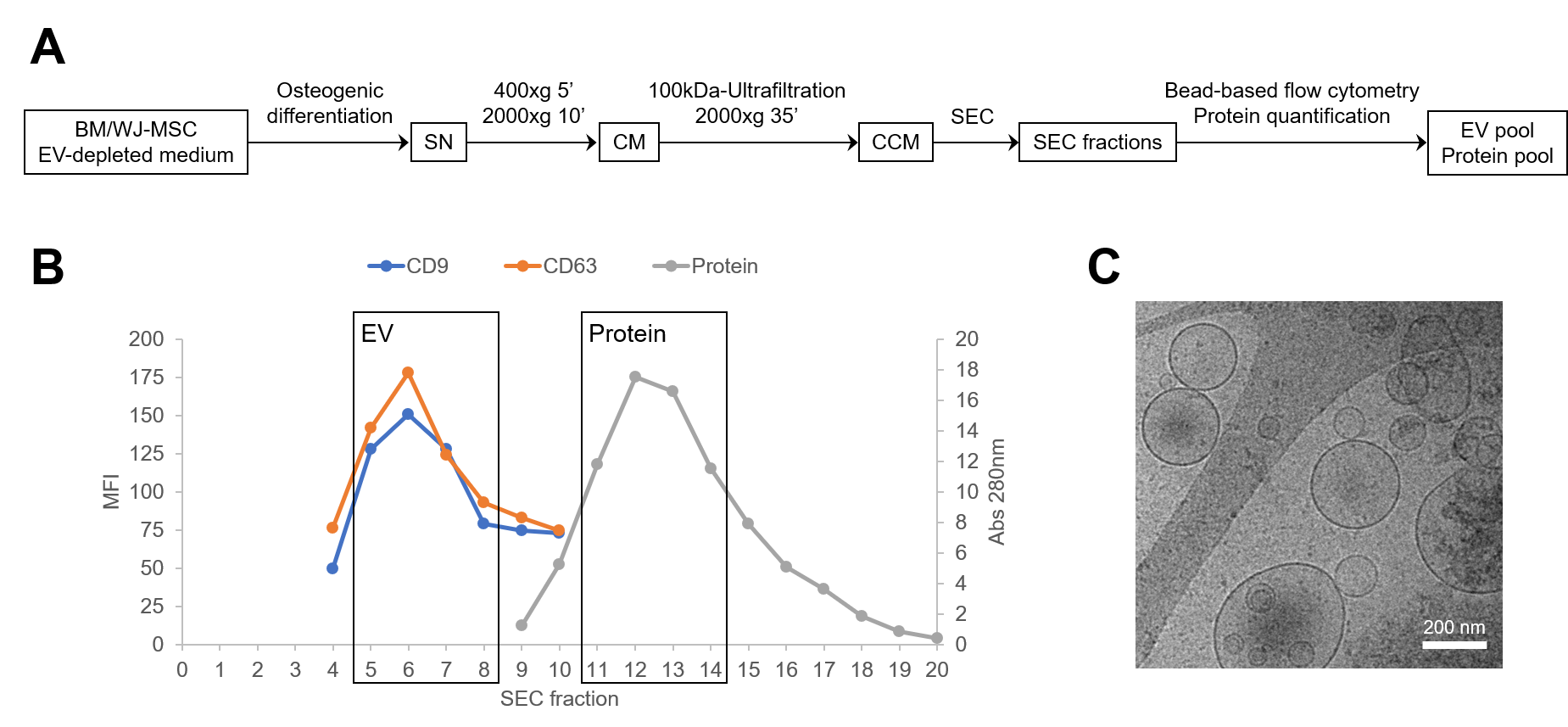


***Additional file* 2. EV and protein fractions isolation and characterization.** (A) Scheme of the methodological procedure followed for EV and protein isolation from BM-MSC and WJ-MSC conditioned media. CCM, concentrated conditioned media; CM, conditioned media; EV, extracellular vesicles; SEC, size exclusion chromatography; SN, cell culture supernatant. (B) Example of representative elution profile obtained for CD9 and CD63 EV markers quantification by bead-based flow cytometry (left axis) and for protein elution monitoring by absorption at 280 nm (right axis) in the different SEC fractions. MFI, mean fluorescence intensity. (C) Cryo-EM images confirming EVs presence in pooled EV fractions. Scale bar: 200 nm.
