# Supplementary material for: Osteogenic commitment of Wharton’s jelly mesenchymal stromal cells: mechanisms and implications for bioprocess development and clinical application
Source: Stem Cell Res Ther. 2019 Nov 28;10:356. doi: 10.1186/s13287-019-1450-3 (PMC6883559; doi:10.1186/s13287-019-1450-3)
Supplement: Supplementary file 2 — Additional file 2. EV and protein fractions isolation and characterization. (A) Scheme of the methodological procedure followed for EV and protein isolation from BM-MSC and WJ-MSC conditioned media. CCM, concentrated conditioned media; CM, conditioned media; EV, extracellular vesicles; SEC, size exclusion chromatography; SN, cell culture supernatant. (B) Example of representative elution profile obtained for CD9 and CD63 EV markers quantification by bead-based flow cytometry (left axis) and for protein elution monitoring by absorption at 280 nm (right axis) in the different SEC fractions. MFI, mean fluorescence intensity. (C) Cryo-EM images confirming EVs presence in pooled EV fractions. Scale bar: 200 nm. [file 13287_2019_1450_MOESM2_ESM.docx]

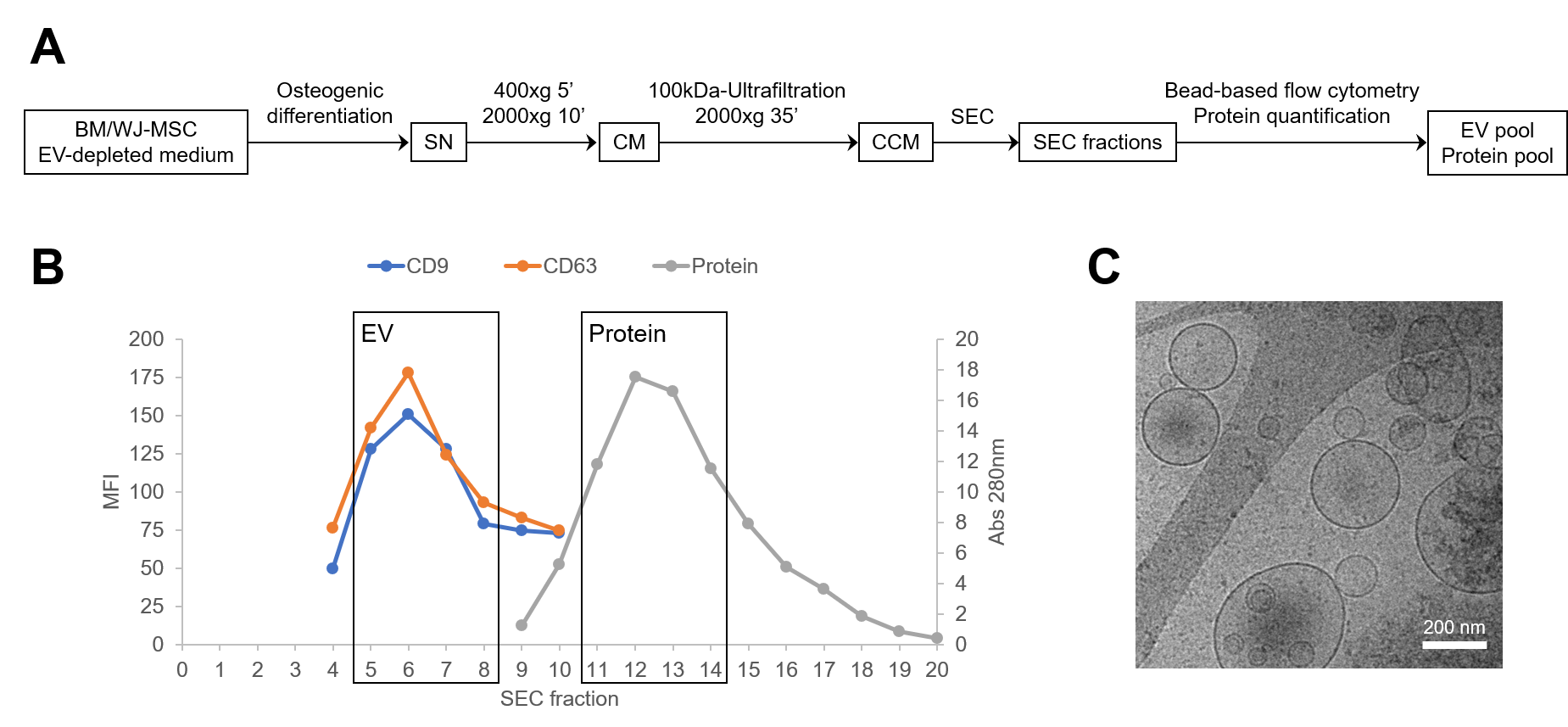


***Additional file* 2. EV and protein fractions isolation and characterization.** (A) Scheme of the methodological procedure followed for EV and protein isolation from BM-MSC and WJ-MSC conditioned media. CCM, concentrated conditioned media; CM, conditioned media; EV, extracellular vesicles; SEC, size exclusion chromatography; SN, cell culture supernatant. (B) Example of representative elution profile obtained for CD9 and CD63 EV markers quantification by bead-based flow cytometry (left axis) and for protein elution monitoring by absorption at 280 nm (right axis) in the different SEC fractions. MFI, mean fluorescence intensity. (C) Cryo-EM images confirming EVs presence in pooled EV fractions. Scale bar: 200 nm.
